# Supplementary material for: Socio-economic factors and its influence on the association between temperature and dengue incidence in 61 Provinces of the Philippines, 2010–2019
Source: PLoS Negl Trop Dis. 2023 Oct 23;17(10):e0011700. doi: 10.1371/journal.pntd.0011700 (PMC10621993; doi:10.1371/journal.pntd.0011700)
Supplement: S1 Table — (DOCX) [file pntd.0011700.s001.docx]

## **S1 Table. Sources of data meta-regressors**

Provincial data for the meta-regressors were all lifted from the 2015 Philippine Census Report by the Philippine Statistics Authority.^1^ These factors included population density, people living in urban areas, average household size, poverty incidence, and health spending.

| **Meta-regressor** | **Year** | **Data source** | **Relevant Literature** |
| --- | --- | --- | --- |
| Population density | 2015 | Philippine Statistics Authority, 2015^1^ | Schmidt et al., 2011; Romeo-Aznar et al., 2022; do Carmo et al., 2020^2–4^ |
| People living in urban areas | 2015 | Philippine Statistics Authority, 2015^1^ | Schmidt et al., 2011; Mutucumarana et al., 2020^2,5^ |
| Average household size | 2015 | Philippine Statistics Authority, 2015^1^ | Da Conceição, et al., 2020; Farinelli et al., 2018 ^6,7^ |
| Poverty incidence | 2015 | Philippine Statistics Authority, 2015^1^ | Da Conceição et al., 2020; Farinelli et al, 2018; do Carmo et al., 2020; Bavia et al., 2020^4,6–8^ |
| Health spending | 2015 | Philippine Statistics Authority, 2015^1^ | Castro et al., 2021; Haakenstad et al., 2022^9,10^ |

## **References**

1. Philippine Statistics Authority. 2015 Census of Population.

2. Schmidt WP, Suzuki M, Dinh Thiem V, White RG, Tsuzuki A, Yoshida LM, et al. Population Density, Water Supply, and the Risk of Dengue Fever in Vietnam: Cohort Study and Spatial Analysis. Farrar J, editor. PLoS Med. 2011 Aug 30;8(8):e1001082.

3. Romeo-Aznar V, Picinini Freitas L, Gonçalves Cruz O, King AA, Pascual M. Fine-scale heterogeneity in population density predicts wave dynamics in dengue epidemics. Nat Commun. 2022 Feb 22;13(1):996.

4. do Carmo RF, Silva Júnior JVJ, Pastor AF, de Souza CDF. Spatiotemporal dynamics, risk areas and social determinants of dengue in Northeastern Brazil, 2014–2017: an ecological study. Infect Dis Poverty. 2020 Dec;9(1):153.

5. Mutucumarana CP, Bodinayake CK, Nagahawatte A, Devasiri V, Kurukulasooriya R, Anuradha T, et al. Geospatial analysis of dengue emergence in rural areas in the Southern Province of Sri Lanka. Trans R Soc Trop Med Hyg. 2020 Jun 1;114(6):408–14.

6. Da Conceição Araújo D, Dos Santos AD, Lima SVMA, Vaez AC, Cunha JO, Conceição Gomes Machado de Araújo K. Determining the association between dengue and social inequality factors in north-eastern Brazil: A spatial modelling. Geospatial Health [Internet]. 2020 Jun 17 [cited 2023 Mar 10];15(1). Available from: https://geospatialhealth.net/index.php/gh/article/view/854

7. Farinelli EC, Baquero OS, Stephan C, Chiaravalloti-Neto F. Low socioeconomic condition and the risk of dengue fever: A direct relationship. Acta Trop. 2018 Apr;180:47–57.

8. Bavia L, Melanda FN, de Arruda TB, Mosimann ALP, Silveira GF, Aoki MN, et al. Epidemiological study on dengue in southern Brazil under the perspective of climate and poverty. Sci Rep. 2020 Feb 7;10(1):2127.

9. Castro M, Mattos E, Patriota F. The effects of health spending on the propagation of infectious diseases. Health Econ. 2021 Sep;30(10):2323–44.

10. Haakenstad A, Coates M, Bukhman G, McConnell M, Verguet S. Comparative health systems analysis of differences in the catastrophic health expenditure associated with non-communicable vs communicable diseases among adults in six countries. Health Policy Plan. 2022 Oct 12;37(9):1107–15.
