# Supplementary material for: Socio-economic factors and its influence on the association between temperature and dengue incidence in 61 Provinces of the Philippines, 2010–2019
Source: PLoS Negl Trop Dis. 2023 Oct 23;17(10):e0011700. doi: 10.1371/journal.pntd.0011700 (PMC10621993; doi:10.1371/journal.pntd.0011700)
Supplement: S2 Table — The corresponding median values of the socio-economic factors were used, depending on the distribution of each dataset. (DOCX) [file pntd.0011700.s002.docx]

**S2 Table. Description of socio-economic factors used for the study**

The socio-economic factors used were province specific. All were originally yearly data, except for latitude. The corresponding median values of the socio-economic factors were used, depending on the distribution of each dataset.

| **Variable** | **Description** |
| --- | --- |
| Poverty incidence | Proportion of people living under the poverty line per 100,000 people in each province yearly between 2010-2019 |
| Population density | Number of people living in each province per square meter, yearly between 2010-2019 |
| Mean number of people per household | Average number of people living in each household, as reported per province, yearly between 2010-2019 |
| People living in urban areas | Percentage of people living in urban areas per 100,000 people in each province between 2010-2019 |
| Health expenditure | Per capita government spending on health in each province, yearly between 2010-2019 |
| Latitude | Mean geographical latitude of each capital location centroid |
