# Supplementary material for: Socio-economic factors and its influence on the association between temperature and dengue incidence in 61 Provinces of the Philippines, 2010–2019
Source: PLoS Negl Trop Dis. 2023 Oct 23;17(10):e0011700. doi: 10.1371/journal.pntd.0011700 (PMC10621993; doi:10.1371/journal.pntd.0011700)
Supplement: S3 Table — (DOCX) [file pntd.0011700.s003.docx]

## **S3 Table**. Main effects of the effect modifiers

| **Effect modifier** | **Relative Risk** | **(95% Confidence Interval)** | **p-value** |
| --- | --- | --- | --- |
| Population Density | 0.997 | (0.996, 0.998) | < 0.001 |
| People living in urban areas | 0.858 | (0.814, 0.904) | < 0.001 |
| Average Household Size | 54.5 | (1.75, 1690) | 0.02 |
| Poverty Incidence | 9.39 x 10^43^ | (8.64 x 10^37^, 1.02 x 10^50^) | < 0.001 |
| Health Spending Per Capita | 0.998 | (0.997, 0.999) | < 0.001 |
| Latitude | 1.26 | (0.879, 1.81) | 0.21 |
