# Supplementary material for: Socio-economic factors and its influence on the association between temperature and dengue incidence in 61 Provinces of the Philippines, 2010–2019
Source: PLoS Negl Trop Dis. 2023 Oct 23;17(10):e0011700. doi: 10.1371/journal.pntd.0011700 (PMC10621993; doi:10.1371/journal.pntd.0011700)

**S1 Fig. Correlation plot between the temperature data from ERA5-land and existing background monitoring stations in Metro Manila and Cebu.**

Statistical correlation of temperature between information from background monitoring stations from the NOAA and ERA5-land was done to confirm the accuracy of the data (Fig. S1). Both results from (A) Metro Manila and (B) Cebu were statistically significant, with a Pearson’s correlation coefficient of 0·88 (p-val < 0·05) and 0·86 (p-val < 0·05), respectively. Abbreviations: “NOAA” = National Oceanic and Atmospheric Administration.
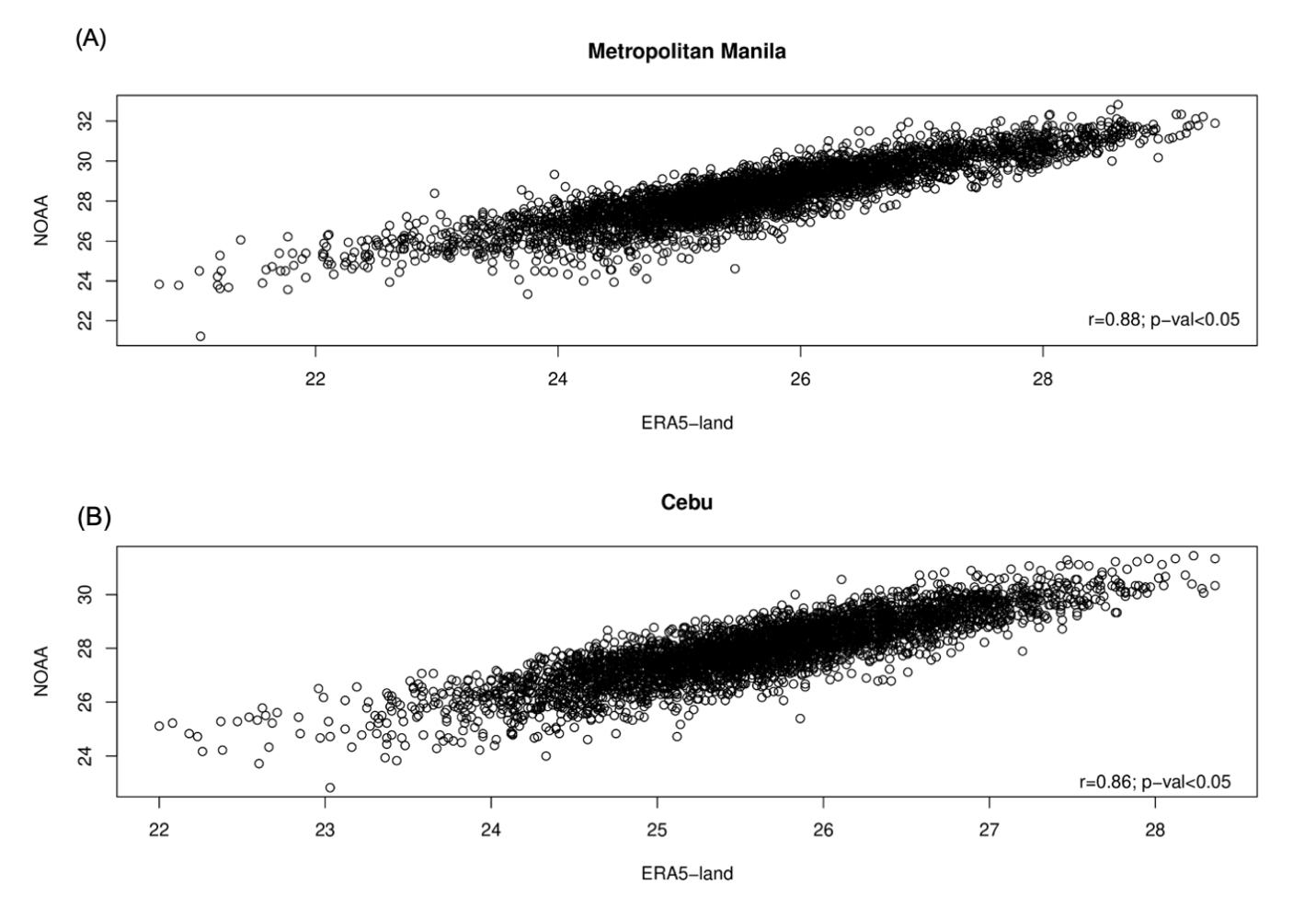

Supplement: S1 Fig — (DOCX) [file pntd.0011700.s004.docx]
