# Supplementary material for: Socio-economic factors and its influence on the association between temperature and dengue incidence in 61 Provinces of the Philippines, 2010–2019
Source: PLoS Negl Trop Dis. 2023 Oct 23;17(10):e0011700. doi: 10.1371/journal.pntd.0011700 (PMC10621993; doi:10.1371/journal.pntd.0011700)

## **S2 Fig. Maximum lag sensitivity analyses**

Maximum Lag 4 (one month) to maximum Lag 48 (4 years) were examined. After changing the maximum lag length for the crude model, (dengue ~ (1|cluster), we took the Akaike Information Criterion (AIC) value for each maximum lag. After generating the AIC values for the whole set of sensitivity values, we implemented a segmented regression analysis to aid in determining the maximum lag. Here, we note that the maximum lag is at 17.5 weeks (approximately 18 weeks; 4.5 months). Abbreviations: “AIC” = Akaike Information Criterion. The blue hollow dot is the 18-week lag selected by segmented regression. The red vertical error bar represents the uncertainty of the 18-week lag. In brief, it means that the central estimate of the maximum lag is 18, with the 95% CI falling between 17 and 19.


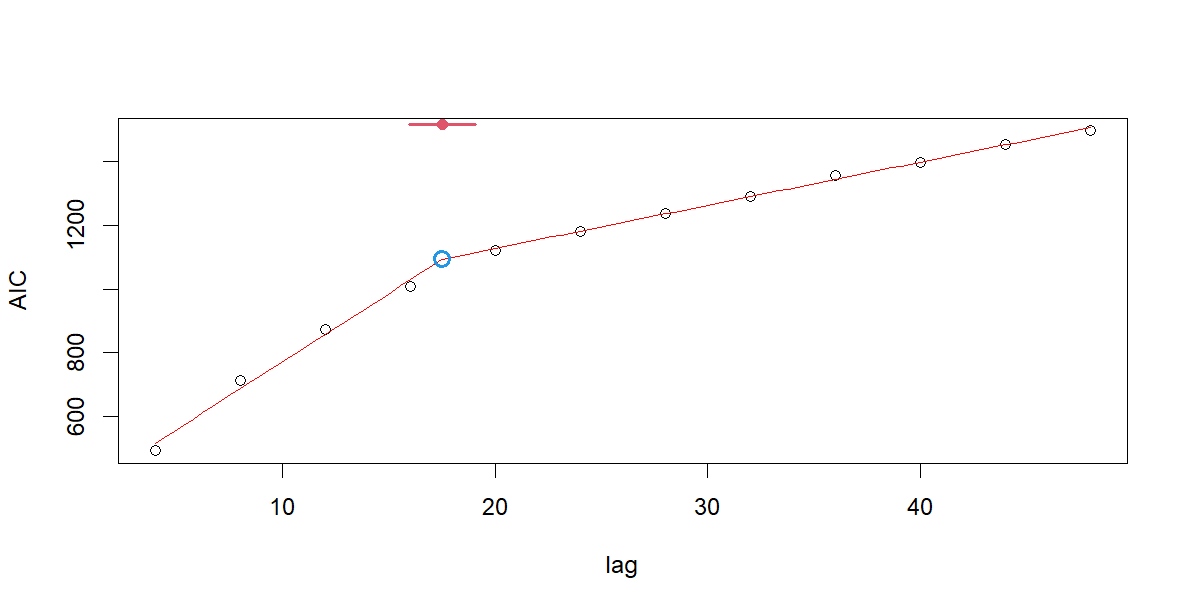

Supplement: S2 Fig — (DOCX) [file pntd.0011700.s005.docx]
