# Supplementary material for: Socio-economic factors and its influence on the association between temperature and dengue incidence in 61 Provinces of the Philippines, 2010–2019
Source: PLoS Negl Trop Dis. 2023 Oct 23;17(10):e0011700. doi: 10.1371/journal.pntd.0011700 (PMC10621993; doi:10.1371/journal.pntd.0011700)

## **S3 Fig. Sensitivity analysis used for df selection**

The sensitivity analysis for df selection for seasonality adjustment showed that the AIC value did not change substantially beyond df 4, thus the choice for the current modeling process. Abbreviations: “QAIC” = Quasi-Akaike Information Criterion


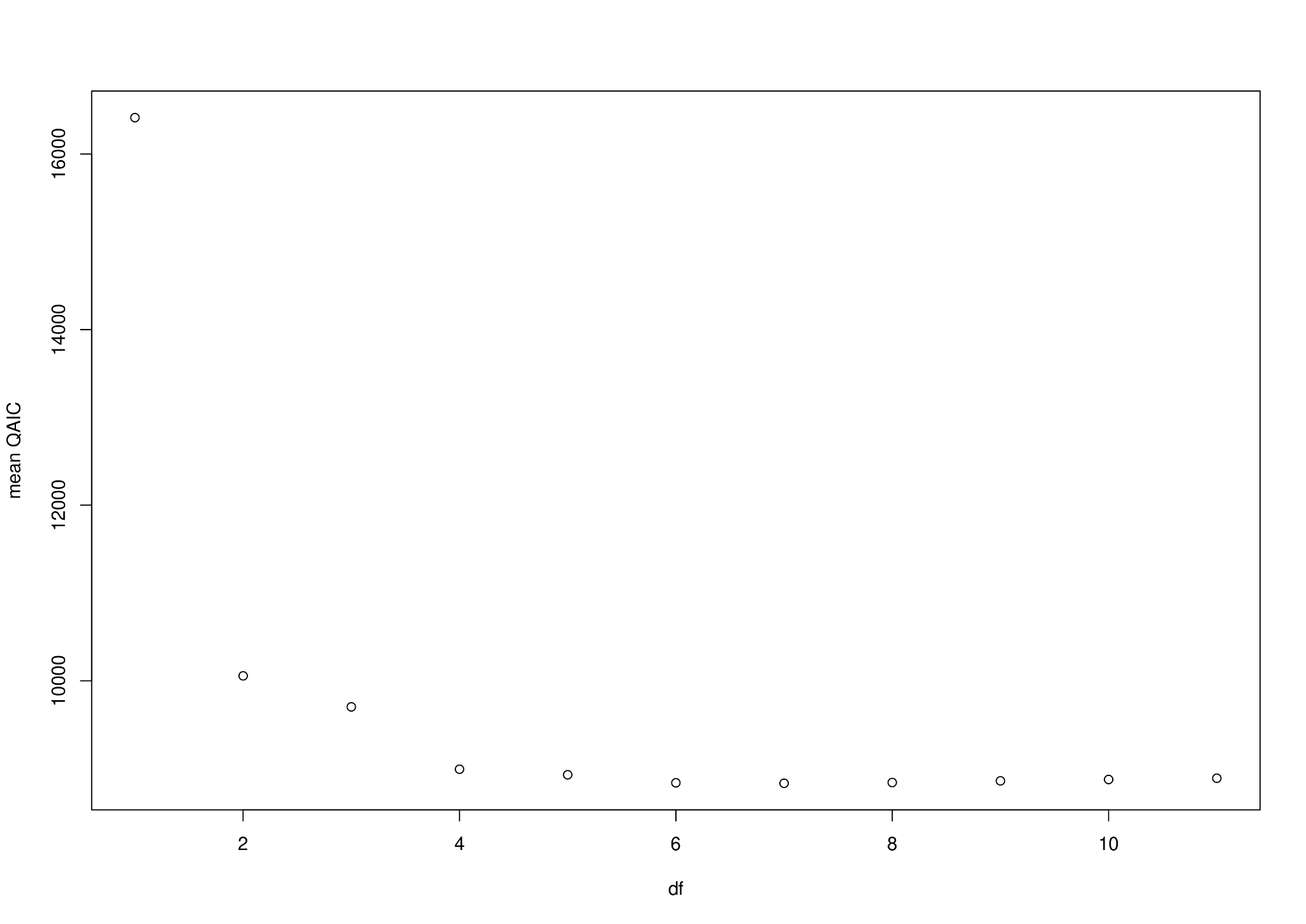

Supplement: S3 Fig — (DOCX) [file pntd.0011700.s006.docx]
