# Supplementary material for: Socio-economic factors and its influence on the association between temperature and dengue incidence in 61 Provinces of the Philippines, 2010–2019
Source: PLoS Negl Trop Dis. 2023 Oct 23;17(10):e0011700. doi: 10.1371/journal.pntd.0011700 (PMC10621993; doi:10.1371/journal.pntd.0011700)

## **S4 Fig. Variation in the annual distribution of cases per year**

Noting the shift of the median value of the dengue case distribution, prompted the use of a random term for year to account for this yearly variation.


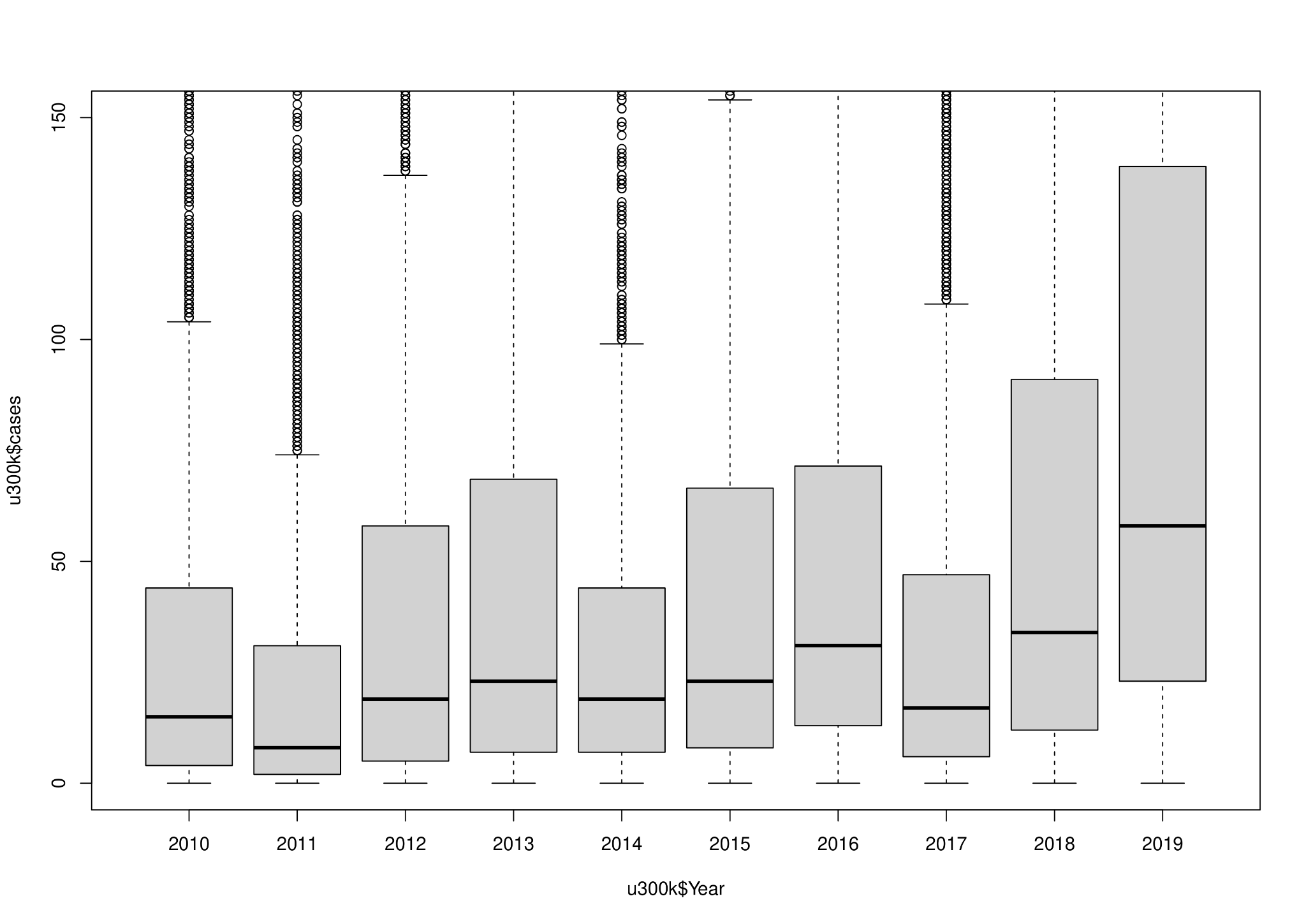

Supplement: S4 Fig — (DOCX) [file pntd.0011700.s007.docx]
