# Supplementary material for: Socio-economic factors and its influence on the association between temperature and dengue incidence in 61 Provinces of the Philippines, 2010–2019
Source: PLoS Negl Trop Dis. 2023 Oct 23;17(10):e0011700. doi: 10.1371/journal.pntd.0011700 (PMC10621993; doi:10.1371/journal.pntd.0011700)

## **S5 Fig. Sensitivity analyses adjusting for precipitation derived from ERA5-land**

Sensitivity analyses adjusting for precipitation derived from ERA5-land showed highly similar risk function, suggesting little to no effect of rainfall on the temperature-dengue association (green line). As there was no significant difference, we opted for the crude model (yellow line). Abbreviations: “RR” = relative risk. Central estimates are shown in solid lines, whereas the 95% Confidence Intervals are shown in the respective shaded region.


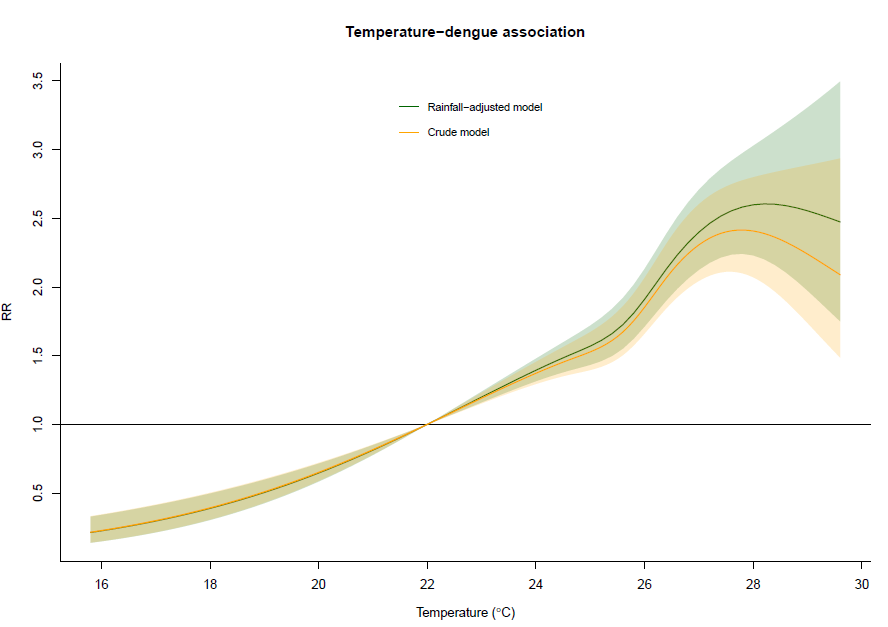

Supplement: S5 Fig — (DOCX) [file pntd.0011700.s008.docx]
