# Supplementary material for: Socio-economic factors and its influence on the association between temperature and dengue incidence in 61 Provinces of the Philippines, 2010–2019
Source: PLoS Negl Trop Dis. 2023 Oct 23;17(10):e0011700. doi: 10.1371/journal.pntd.0011700 (PMC10621993; doi:10.1371/journal.pntd.0011700)

## **S6 Fig. Temperature-specific associations in different temperature percentiles**

The temperature-specific associations have been calculated at lag 0 (A), lag 3 (B), lag 6 (C), lag 9 (D), lag 12 (E), lag 18 (F). We have included the temperature-specific associations in the 1st (20.8 degC) (G), 5th (23 degC) (H), 10th (23.6 degC) (I), 90th (27 degC) (J), 95th (27.6 degC) (K) and 99th (28 degC) (L) temperature percentiles in Figure S6 (right panel). All of the lag patterns in various temperature percentiles peaks at around 16 weeks, except for the 1st percentile (20.8 degC). Central estimates are shown in solid black line, whereas the 95% Confidence Intervals are shown in grey-shaded region.


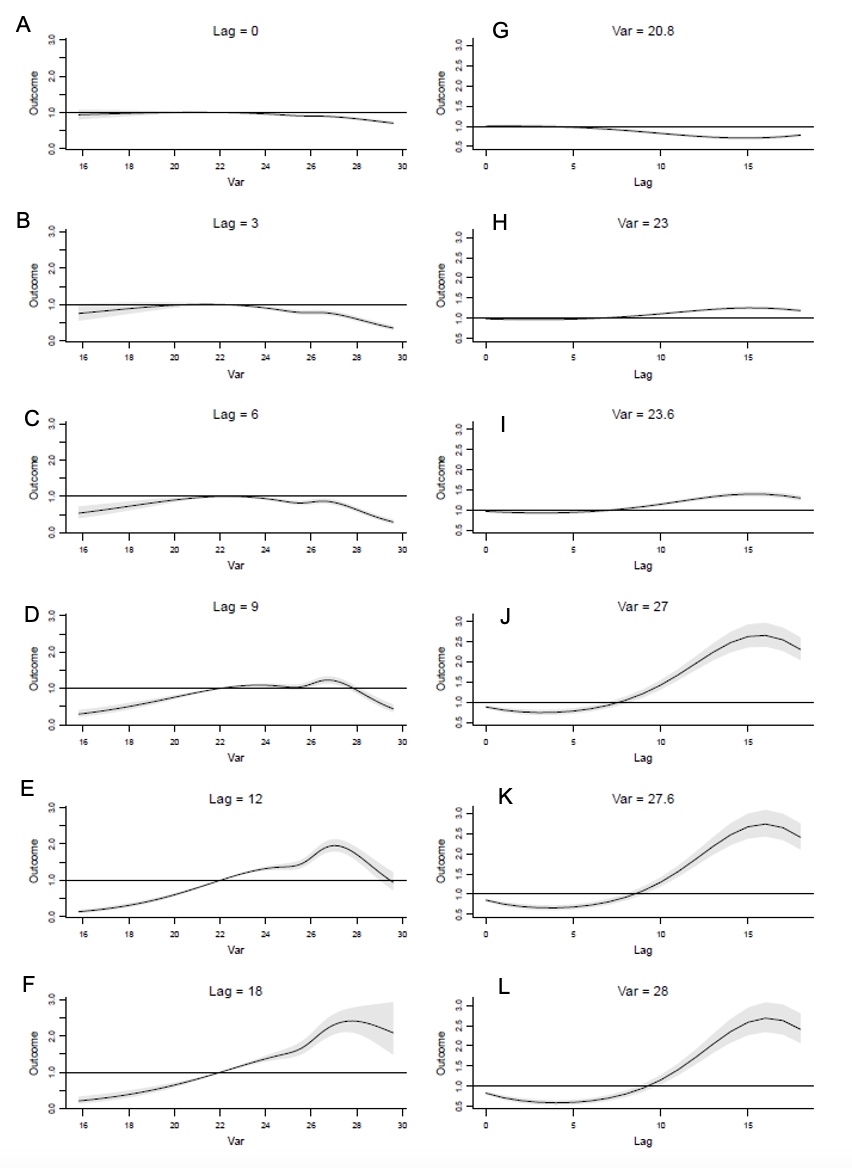

Supplement: S6 Fig — (DOCX) [file pntd.0011700.s009.docx]
