# Supplementary material for: Socio-economic factors and its influence on the association between temperature and dengue incidence in 61 Provinces of the Philippines, 2010–2019
Source: PLoS Negl Trop Dis. 2023 Oct 23;17(10):e0011700. doi: 10.1371/journal.pntd.0011700 (PMC10621993; doi:10.1371/journal.pntd.0011700)
Supplement: S7 Fig — (DOCX) [file pntd.0011700.s010.docx]

**S7 Fig. Interaction analyses per effect modifier**

This figure depicts the modifying effect of each socio-economic variable on the dengue-temperature association, including population density (A), people living in urban areas (B), average household size (C), poverty incidence (D), health spending per capita (E), and latitude (F). Central estimates are shown in solid black line, whereas the 95% Confidence Intervals are shown in grey-shaded region. Y-axis represents the ratio of relative risk (RRR) whereby the comparator risk is assumed as at the 5^th^ temperature percentile. X-axis is temperature.

## **
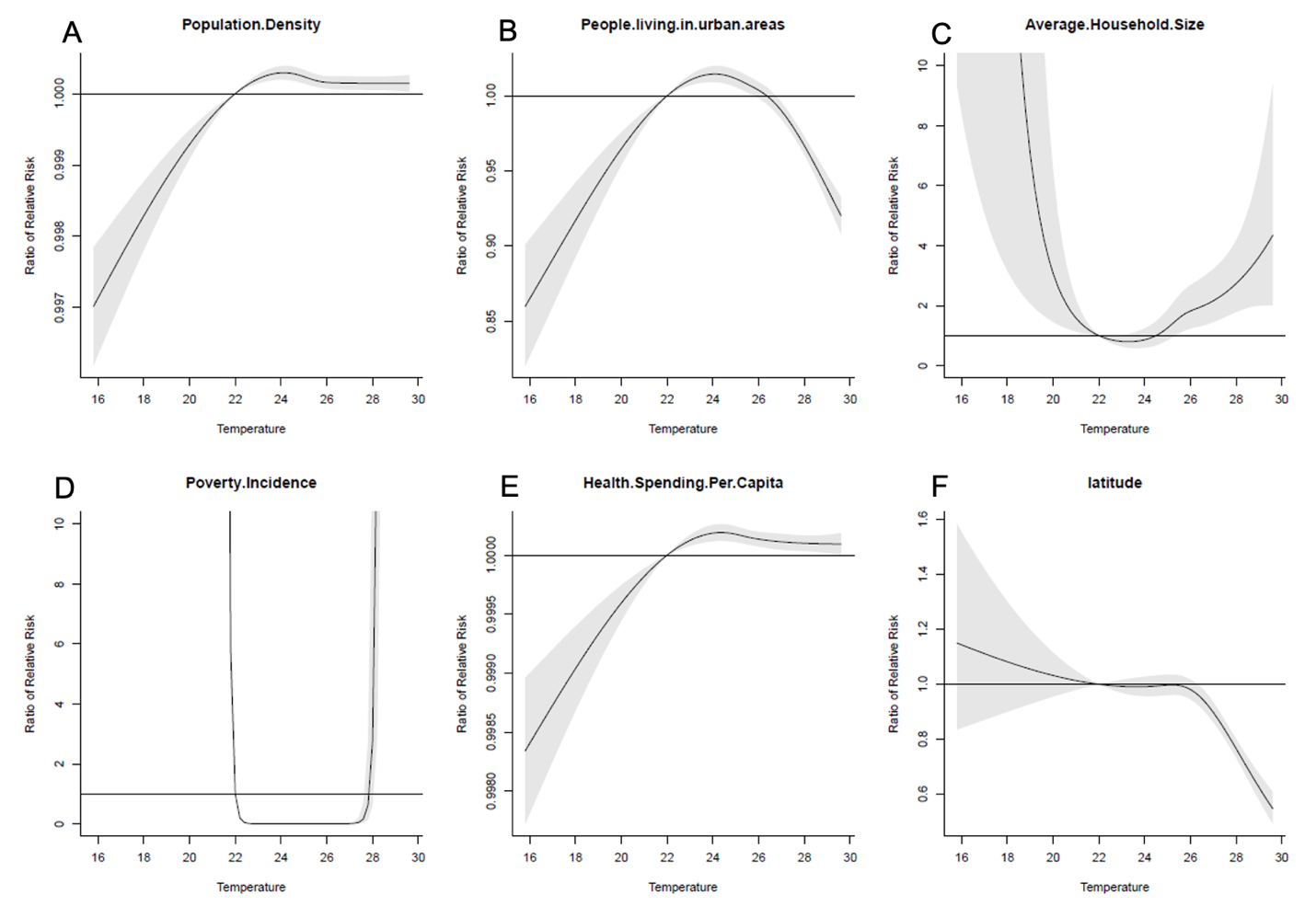
**
