# Supplementary material for: Socio-economic factors and its influence on the association between temperature and dengue incidence in 61 Provinces of the Philippines, 2010–2019
Source: PLoS Negl Trop Dis. 2023 Oct 23;17(10):e0011700. doi: 10.1371/journal.pntd.0011700 (PMC10621993; doi:10.1371/journal.pntd.0011700)
Supplement: Attachment — Submitted filename: PlosNTDs_dengue_temp_SES.docx [file pntd.0011700.s011.docx]

In this study, authors examine how various socio-economic factors (including poverty, urbanization, and health-care expenditures) modify the temperature-dengue incidence across provinces in Philippines. They do so using a two-stage distributed lag non-linear model with meta-analysis. This research question is of importance, and the DLNM model is a good approach for this question.

After reading the manuscript, I have outstanding questions about model specification as well as several concerns with the model results and interpretation. My major concerns are as follows:

1. The **parameterization of several meta-predictors** are unclear to me, and should be adjusted, as follows:
   a. Poverty incidence – is this the same as proportion of individuals living beneath the poverty line?
   b. Number of people per household – is this the *mean* number of people per household?
   c. Health expenditure – is this total expenditures or per capita? The latter would make more sense to me as a predictor. The former may introduce concerns that the predictor is simply reflecting province size.
   d. People living in urban areas – is this the total number of people living in urban areas or the percentage of people living in urban areas? The latter would make more sense to me as a predictor. The former may introduce concerns that the predictor is simply reflecting province size.
2. **Association between temperature and dengue incidence** (exposure-response dimension; lines 169-172, Figure 3): The temperature-dengue incidence is not described at any particular lag. For what lag are authors describing this relationship? Does the relationship chance over different lags?
3. **Association between temperature and dengue incidence** (lag-response dimension; lines 174-174, Figure 4): The lag-response relationship is not described at any particular temperature comparison. For what temperature comparison are authors describing this relationship? Does the lag-response relationship different for other temperature comparisons?
4. **Introduction: effect modification by socio-economic factors** – The key premise of the paper is examining whether the climate-dengue relationship is stronger among certain vulnerable sub-populations. In the introduction, the authors should provide more literature around why it is reasonable to hypothesize that climate-disease relationships are stronger among certain sub-populations. Even if it is not available for dengue, there is literature for other climate-health outcomes (e.g., for heat-mortality relationship) to show that disadvantaged communities have lower adaptive capacity and/or are more susceptible to negative health outcomes when exposed to environmental hazards.
5. **Results: effect modification by socio-economic factors** – As shown in Figure 5, I find it curious why the temperature-response relationship is highly uncertain for one SES axes of comparison, but not the other. Do the authors understand why there would be substantial uncertainty in the temperature-response relationship among provinces with low population density, say, but not high population density? The high uncertainty on the poverty incidence comparison could also be leading to the null finding of effect medication, as the temperature-response curves are quite different for the two, but there is so much uncertainty around the high poverty incidence line. As an alternative model specification to the meta-analysis, authors may consider interacting the crossbasis term in the national model (equation 2) by the dichotomous SES variable of interest. Doing so would also give the benefit of being able to interpret the SES variable as a main effect (see point 7).
6. **Results: effect modification by socio-economic factors** – In the results (line 179-184), the authors should go beyond saying which factors were effect modifiers, and describe how temperature-dengue relationships vary by SES strata. For instance, the authors could describe how dengue incidence within provinces with low healthcare expenditures is highly sensitive to changes in temperature, while dengue incidence provinces with high healthcare expenditures is less sensitive.
7. **Discussion**: **effect modification by socio-economic factors** – In the discussion (lines 208-255), the authors should take care not to confuse interpretation of effect modifiers and main effects. For instance, the results provided do not say anything about the relationship between poverty and dengue incidence, and thus do not contrast with existing literature finding an association between poverty and dengue. Poverty incidence would have to be included as a main effect in the model in order to make that comparison. Instead, poverty incidence was not found to be a significant effect modifier of the temperature-dengue association (although, see point 5 about uncertainty).
8. **Additional model confounders:** while the inverted U-shaped relationship with temperature makes sense, I am curious if the authors considered adjusting for precipitation as a confounder, as precipitation is known to influence dengue, and also be associated with temperature.

Additionally, I have some minor concerns that may improve the readability of the manuscript:

Abstract:

- “role of socio-economic on the temperature-dengue association remains unclear” could be phrased better (e.g., “heterogeneity in the temperature-dengue relationship across axes of socio-economic vulnerability is not well described.”
- “explore the modification role by socioeconomic factors” could be edited to “explore effect modification by socioeconomic factors”
- Keep consistent socio-economic vs. socioeconomic
- Line 34: “analysis in examining” change to “analysis to examine”, change “explain” to past tense
- Line 35: “nation wide heterogeneity” – do you mean “heterogeneity between provinces”?

Introduction

- Line 55 – italicize vector name
- Line 56 – is the 30-fold increase globally?
- Line 65 – change “higher” to “high”
- Lines 74 – 78: are these relationships inverse U-shaped?
- Line 82: add bolded text “linear association **between temperature and dengue**”

Methods

- Line 97: change to “and were thus excluded from the current study”
- Line 99: what is the spatial resolution of ERA5-land? Also cite ERA5-land data
- Line 102: remove word “significantly”
- Line 104: add “Over the same period,…”
- Line 114: I would describe this as a DLNM with a negative binomial distribution on the outcome rather than a negative binomial regression coupled with a DLNM
- Add equation numbers to equations 1 and 2
- Lines 124-126: what kind of splines were used?
- Line 129 and Equation 1: I believe the term should be Y­_(t-1,i) instead of Y_(t, i-1). Also the error term symbol is missing in line 129 on my version.
- Lines 135 – 136: Is the pooled association obtained through equation 2 or by using meta-analysis to pool?
- Line 136: Instead of “heterogeneity in the pooled association”, do you mean “heterogeneity in the province-specific associations”?
- Line 138 – 140: citations needed for the packages

Results

- Line 147: Is the median and range for cases per year? Per week?
- Lines 146 – 150: Only report the ranges and medians for the 61 provinces
- Line 148: can get by with fewer decimals on temperatures
- Figure 1: In the downloadable figure, it looks OK, but in the in-text figure, A and B panels are different sizes. Ensure they are similarly sized.

Discussion

- Line 191: The text here says 27.1 but earlier it said 27.2
- Line 221: “It’s vectors” – name the vectors
- Limitations: could some of the SES variables studied affect reporting? For instance, some discussion could be provided around the fact that areas with higher poverty may be less likely to report dengue incidence, thus biasing relationships towards the null
- Line 261: “decentralized” may be a better term than “devolved”
