# Supplementary material for: Socio-economic factors and its influence on the association between temperature and dengue incidence in 61 Provinces of the Philippines, 2010–2019
Source: PLoS Negl Trop Dis. 2023 Oct 23;17(10):e0011700. doi: 10.1371/journal.pntd.0011700 (PMC10621993; doi:10.1371/journal.pntd.0011700)
Supplement: Attachment — Submitted filename: Review for PNTD-D-23-00460.docx [file pntd.0011700.s012.docx]

Review for “Socio-economic factors and its influence on the association between temperature and dengue incidence in 61 Provinces of the Philippines”.

This paper used DLNM to identify the association between temperature and dengue incidence by each of the 61 provinces of the Philippines. The paper then conducted meta regression to identify how socioeconomic conditions (as meta regressors) modified these associations. Overall, I think the manuscript is well written and the research design is fine.

Here are some additional comments that the authors may want to address:

1. Line 113, the two-stage analysis (i.e. provincial DLMN + meta regression) is self-coherent. However, I don’t see the need for analyzing the nationwide temperature-dengue associations. It might be misleading for the readers to think that an analysis using national-level time-series of temperature and dengue cases was conducted.

2. The choice of certain modeling parameters, such as df=4 for temperature, needs to be justified. In addition, while the authors justified the 18-week maximum time lag based on sensitivity analysis (Figure S2), whether such a lag was supported by the disease process can be explained in the manuscript.

3. Line 147, is the “median of 23, and interquartile range of 57” for cases per epiweek?

4. Line 171, the meaning of I2 and how it determines the heterogeneity can be explained in the manuscript.

5.Line 203-204, the statement that 11 weeks accounts for the full cycle of the disease process from mosquito hatching to incubation in infected humans needs some reference.

6. Line 205, “countries” should be “locations”, as Bangkok is not a country.

7. Figure 3, I found it interesting that all the provincial temperature-relative risk curves indicated RR=1 at temperature=25 degrees C. Is there any explanation for this?

8. Also Figure 3 shows that in some provinces RR decreases as temperature gets warmer till 25 degree C, which is in the contrary to the statement between L169-171. Could the authors address/explain this?

9. Lastly, such a two-stage analysis is also used (quite sometimes) in temperature-mortality studies (e.g. Bakhtsiyarava, M., et al.. (2023). Modification of temperature-related human mortality by area-level socioeconomic and demographic characteristics in Latin American cities. Social Science & Medicine, 115526.). The authors could provide such context in the manuscript.
