# Supplementary material for: Socio-economic factors and its influence on the association between temperature and dengue incidence in 61 Provinces of the Philippines, 2010–2019
Source: PLoS Negl Trop Dis. 2023 Oct 23;17(10):e0011700. doi: 10.1371/journal.pntd.0011700 (PMC10621993; doi:10.1371/journal.pntd.0011700)
Supplement: Attachment — Submitted filename: PLOS NTD Responses to Reviewers.docx [file pntd.0011700.s013.docx]

18 Aug 2023

**Qu Cheng, Ph.D.**

*Guest Editor*

PLOS Neglected Tropical Diseases

**Justin Remais**

*Section Editor*

PLOS Neglected Tropical Diseases

**To Dr. Cheng and Mr. Remais:**

We would like to express our sincere gratitude for considering our manuscript entitled **"Socio-economic factors and its influence on the association between temperature and dengue incidence in 61 Provinces of the Philippines, 2010-2019"** for publication in your esteemed journal. We greatly appreciate the time and effort invested by the reviewers in providing valuable feedback that has helped us improve the quality of our work.

We are pleased to submit our detailed response to the reviewers' comments, which we believe addresses all their concerns and suggestions. Our response document includes point-by-point explanations of the revisions made, along with corresponding page and line numbers for easy reference. We have also attached the revised manuscript, clearly indicating the modifications made using track changes. The revisions made to the manuscript are aimed at enhancing the clarity, scientific rigor, and overall quality of the study. We have carefully addressed each comment and incorporated appropriate changes to ensure the manuscript aligns with the high standards set by PLOS Neglected Tropical Diseases.

We hope that the reviewers and the editorial board find our responses and revisions satisfactory and deem the manuscript suitable for the next steps toward publication. Once again, we extend our deepest appreciation to the editors and reviewers for their time, expertise, and invaluable feedback. We are confident that the revisions made have substantially improved the manuscript, and we hope for a positive outcome in the final evaluation. Please feel free to contact us if any further information or clarification is required.

Thank you very much for your kind consideration.

Sincerely,

**XERXES T. SEPOSO**

*Associate Professor*

Hokkaido University

Sapporo, Japan

**Authors’ Responses to Reviewers’ Questions**

Reviewer 1: Blue

Reviewer 2: Red

Reviewer 3: Green

**From General Email**

Key Review Criteria Required for Acceptance?

As you describe the new analyses required for acceptance, please consider the following:

Methods

-Are the objectives of the study clearly articulated with a clear testable hypothesis stated?</br>

-Is the study design appropriate to address the stated objectives?</br>

-Is the population clearly described and appropriate for the hypothesis being tested?</br>

-Is the sample size sufficient to ensure adequate power to address the hypothesis being tested?</br>

-Were correct statistical analysis used to support conclusions?</br>

-Are there concerns about ethical or regulatory requirements being met?</br></br>

Reviewer #1: I document major concerns in the attached. I am concerned about the omission of precipitation in the model, and in the parameterization of certain socioeconomic variables (e.g., is urban population used or percent urban population used?)

The authors’ thank Reviewer 1 for their comments and suggested revisions. The responses to each comment of the attached document of the reviewer have been addressed in the document below under R1-Document.

Reviewer #2: 1. The objectives of the study clearly articulated with a clear testable hypothesis stated - 1. to examine the association between dengue and temperature in multiple locations in the Philippines, and 2. to explore the modification role by socioeconomic factors.

No question was made.

2. The study design is appropriate to address the stated objectives - a modelling analysis has been carried out.

No question was made.

3. The population is clearly described and appropriate for the hypothesis being tested.

No question was made.

4. Correct statistical analysis were used to support conclusions - meta-regression is common to be used to summarize the effect size of the association.

No question was made.

5. There are concerns about ethical or regulatory requirements being met - No ethical statement has been mentioned.

The authors’ have included an Ethical statement in the revised manuscript in line 332-335. “The Ateneo de Manila University Research Ethics Committee waived the need for ethical approval and the need to obtain consent for collection, analysis, and publication of secondarily, retrospectively obtained for this non-interventional study. All methods were performed in accordance with the relevant guidelines and regulations.”

Reviewer #3: Yes to all these questions.

No question was made.

--------------------

Results

-Does the analysis presented match the analysis plan?</br>

-Are the results clearly and completely presented?</br>

-Are the figures (Tables, Images) of sufficient quality for clarity?</br></br>

Reviewer #1: I document major concerns in the attached. I am concerned that the authors do not specific over which lags the exposure-response dimensions are calculated, nor for what temperature comparison the lag response dimension is calculated. I am also concerned about why the temperature-response relationship is highly uncertain for one SES axes of comparison, but not the other.

The authors’ thank Reviewer 1 for their comments and suggested revisions. The responses to each comment of the attached document of the reviewer have been addressed in the document below under R1-Document.

Reviewer #2: Yes

No question was made.

Reviewer #3: I have a question regarding Figure 3 and the description about it in the manuscript. I raised this question in my reviewer attachment.

The authors’ thank Reviewer 3 for their comments and suggested revisions. The responses to each comment of the attached document of the reviewer have been addressed in the document below under R3-Document.

--------------------

Conclusions

-Are the conclusions supported by the data presented?</br>

-Are the limitations of analysis clearly described?</br>

-Do the authors discuss how these data can be helpful to advance our understanding of the topic under study?</br>

-Is public health relevance addressed?</br></br>

Reviewer #1: I document major concerns in the attached. The authors should take care not to confuse interpretation of effect modification with main effects. Such is the case, for instance, in poverty incidence.

The authors’ thank Reviewer 1 for their comments and suggested revisions. The responses to each comment of the attached document of the reviewer have been addressed in the document below under R1-Document.

Reviewer #2: Yes

No question was made.

Reviewer #3: Yes to all.

No question was made.

--------------------

<b>Editorial and Data Presentation Modifications?</b></br><br/>

Use this section for editorial suggestions as well as relatively minor modifications of existing data that would enhance clarity. If the only modifications needed are minor and/or editorial, you may wish to recommend “Minor Revision” or “Accept”.

Reviewer #1: (No Response)

No question was made.

Reviewer #2: (No Response)

No question was made.

Reviewer #3: I recommend minor revision.

No question was made.

--------------------

Summary and General Comments

Use this section to provide overall comments, discuss strengths/weaknesses of the study, novelty, significance, general execution and scholarship. You may also include additional comments for the author, including concerns about dual publication, research ethics, or publication ethics. If requesting major revision, please articulate the new experiments that are needed.

1.5.R1. Reviewer #1: In this study, authors examine how various socio-economic factors (including poverty, urbanization, and health-care expenditures) modify the temperature-dengue incidence across provinces in Philippines. They do so using a two-stage distributed lag non-linear model with meta-analysis. This research question is of importance, and the DLNM model is a good approach for this question. However, I have some methodological questions as well as some concerns with the presentation and interpretation of the results.

No question was made. Comments are addressed in the subsequent sections.

Reviewer #2: This is a modelling study 1. to examine the association between dengue and temperature in multiple locations in the Philippines, and 2. to explore the modification role by socioeconomic factors. Some comments:

1.5.R2-1. 1. According to the journal policy, the data are required to be shared to ensure a reproducibility.

The data availability statement has been modified in Line 340-346, as advised:

“The data set for dengue incidence was acquired with permission from the Philippines Department of Health Epidemiological Bureau, while that of the provincial mean weekly temperatures were acquired from the Philippine Atmospheric, Geophysical and Astronomical Services Administration, supplemented by ERA5-land data, which is publicly accessible. The data sets may be accessed upon formal request from the corresponding author or by contacting the data source owners directly at nec.doh@gmail.com +63 [(02) 8651 7800](https://www.google.com/search?q=epidemiology+bureau+contact+number&rlz=1C5CHFA_enPH983PH985&ei=4TJ8ZKmrMu7X2roPy-OtuAk&ved=0ahUKEwjpn5n7gan_AhXuq1YBHctxC5cQ4dUDCA8&uact=5&oq=epidemiology+bureau+contact+number&gs_lcp=Cgxnd3Mtd2l6LXNlcnAQAzIGCAAQBxAeOgoIABBHENYEELADOggIABCKBRCGA0oECEEYAFDrAViABWCaCmgBcAF4AIABfYgB3gOSAQMwLjSYAQCgAQHAAQHIAQg&sclient=gws-wiz-serp) and (+632)8927-1541 or [information@pagasa.dost.gov.ph](mailto:information@pagasa.dost.gov.ph) respectively. R codes to perform the analyses are available upon formal request from the corresponding author.”

2. The study findings are highly consistent with some others in South Asian settings that have not been cited e.g. doi.org/10.1016/j.jiph.2017.12.006, doi.org/10.1016/j.envint.2022.107518. Please discuss.

The authors thank the reviewer for their suggestions and have added these references to the study, as suggested. These can be seen in the following lines:

Line 219-221: “Another study by Servadio, et. al (2017), presented similar results of a similar parabolic curve, with maximum development rate in vector-borne diseases at temperatures between 28 and 32 °C and none under 18C or over 34 °C in South and Southeast Asian countries.”

Line 65-66: “…or even a shift shifts in geographic regions affected by vector-borne disease outbreaks.”

Lines 216-218: “Dengue fever risk was seen to increase after extremely high temperatures due to accelerated incubation and increased mosquito population, although dengue lifespan and egg survival rate was decreased.”

3. Please describe what is "ERA5-land"? Is the data source reliable?

We have modified the manuscript to give a background and the accuracy of ERA5-land, now found in Lines 103-105.

“Developed by the Copernicus Climate Change Service (C3S) on behalf of the European Union, ERA5-land which is a publicly available enhanced global dataset that has shown accurate temperature readings in Europe and Central Asia (Muñoz-Sabater, 2021).”

4. What is the resolution of the socioeconomic factors?

The resolution of the socioeconomic factors can be found in Line 115-117: “The socio-economic factors used were province specific. All were originally yearly data, except for latitude. The corresponding median values of the socio-economic factors were used, depending on the distribution of each dataset. These terms have been operationalized as seen in Table S2.”

5. Maximum lag of 18 weeks is too long even lag plot may support the assumption. Justification should be given.

Lines 225-236: Our results have shown a 16-week (or approximately 4 months) lag association between temperature and dengue incidence as shown in Figure 4. Previous studies have also utilized longer lag periods to examine the lag association. In Singapore, Hii et al. (2009) utilized a 20-week maximum lag to examine the effects of weather variables (including temperature) on dengue incidence. Results showed higher relative risks of dengue cases at a time lag of 3–4 months, which is consistent with our study findings. Similarly, Hii et al. (2012) also utilized a 20-week maximum lag in examining the optimal lead time for dengue forecasting (parameterized by weather variables), with an optimal lead time at around 3 months. A similar study by Koh et al. (2008) found that dengue incidence in 2005 was highly associated with weekly mean temperature at a lag time of 18 weeks. Heng et al. (1998) noted that dengue incidence occurred at a lag time of 8–20 weeks following elevated temperature. Other longer lag associations have been observed in Guadaloupe (Gharbi, et. al, 2011), Indonesia (Astuti et al., 2019), and Malaysia (Jayaraj et al., 2019) at 11-weeks, 12-weeks, and 24-months, respectively. The delayed association between temperature and dengue incidence may potentially be related to the incubation periods in host–vector–pathogen transmissions cycle plus reproduction, maturation and survival rate of vector (Hii et al., 2009).

6. A symbol cannot be showed in line 129.

The manuscript has been modified and can now be seen in Line 140.

7. The first paragraph of results should report the incidence rate rather than number of cases.

The manuscript has been modified as suggested, as shown in Line 166-167, reading “A mean, weekly dengue incidence rate of 2.128 was recorded from 2010-2019 across 61 provinces in the Philippines with a range from 0 to 133.925 per EpiWeek.”

8. The major concern of the study is that the uncertainty of categories is very large in the socioeconomic factors (Figure 5), so the results of the effect modification is very inconclusive. Even household size i don't think it is a effect modifier given both groups show a similar pattern. The authors should think of other ways to examine the modification effect.

This is well noted. In reference to the suggestion of the reviewer. We have implemented an interaction analysis to assess the effect modification by SES. Please refer to the extensive response in point 5.

Reviewer #3: (No Response)

No question was made.

--------------------

PLOS authors have the option to publish the peer review history of their article (<a href="https://journals.plos.org/plosntds/s/editorial-and-peer-review-process#loc-peer-review-history" target="_blank">what does this mean?</a>). If published, this will include your full peer review and any attached files.<br><br>

If you choose “no”, your identity will remain anonymous but your review may still be made public.<br><br>

<b>Do you want your identity to be public for this peer review?</b> For information about this choice, including consent withdrawal, please see our <a href="https://www.plos.org/privacy-policy" target="_blank">Privacy Policy</a>.

Reviewer #1: No

No question was made.

Reviewer #2: No

No question was made.

Reviewer #3: No

No question was made.

—-----------------------

In this study, authors examine how various socio-economic factors (including poverty, urbanization, and health-care expenditures) modify the temperature-dengue incidence across provinces in Philippines. They do so using a two-stage distributed lag non-linear model with meta-analysis. This research question is of importance, and the DLNM model is a good approach for this question.

After reading the manuscript, I have outstanding questions about model specification as well as several concerns with the model results and interpretation. My major concerns are as follows:

1.	The parameterization of several meta-predictors are unclear to me, and should be adjusted, as follows:

a.	Poverty incidence – is this the same as proportion of individuals living beneath the poverty line?

b.	Number of people per household – is this the mean number of people per household?

c.	Health expenditure – is this total expenditures or per capita? The latter would make more sense to me as a predictor. The former may introduce concerns that the predictor is simply reflecting province size.

d.	People living in urban areas – is this the total number of people living in urban areas or the percentage of people living in urban areas? The latter would make more sense to me as a predictor. The former may introduce concerns that the predictor is simply reflecting province size.

The authors recognize the confusion of these terms and have operationalized them under Table S2.

**Table S1.** Description of socio-economic factors used for the study.

| **Variable** | **Description** |
| --- | --- |
| Poverty incidence | Proportion of people living under the poverty line in each province yearly between 2010-2019 |
| Population density | Number of people living in each province per square meter, yearly between 2010-2019 |
| Mean number of people per household | Average number of people living in each household, as reported per province, yearly between 2010-2019 |
| People living in urban areas | Percentage of people living in urban areas in each province between 2010-2019 |
| Health expenditure | Per capita health spending by each province, yearly between 2010-2019 |
| Latitude | Mean geographical latitude of the centroid of the capital location controid |

2.	Association between temperature and dengue incidence (exposure-response dimension; lines 169-172, Figure 3): The temperature-dengue incidence is not described at any particular lag. For what lag are authors describing this relationship? Does the relationship chance over different lags?

Figure 3 depicts the cumulative effect from lags 0 to 18 per temperature. We have included the lag-specific associations for the following weeks (Weeks 0, 3, 6, 9, 12 and 18) in Figure S6 (left panel).

The shape of the exposure-response association remains to be an inverted U relationship pattern, but is less pronounced yet discernable at Lag 0.

3.	Association between temperature and dengue incidence (lag-response dimension; lines 174-174, Figure 4): The lag-response relationship is not described at any particular temperature comparison. For what temperature comparison are authors describing this relationship? Does the lag-response relationship different for other temperature comparisons?

Figure 4 depicts the lag-response curve at the 90^th^ temperature percentile.

We have included the temperature-specific associations in the 1st (20.8 degC), 5th (23 degC), 10th (23.6 degC), 90th (27 degC), 95th (27.6 degC) and 99th (28 degC) temperature percentiles in Figure S6 (right panel). All of the lag patterns in various temperature percentiles peaks at around 16 weeks, except for the 1st percentile (20.8 degC). One of the potential reasons on why the risk went down even after longer lags at the lower temperature period may be due to the pathophysiology of the mosquito and of viral viability. In Guangzhou, China, Xiang et al. (2017) noted that the optimal maximum temperature range for dengue transmission was 21.6 to 32.9 degC. The first percentile temperature (at 20.8 degC) may prove to be suboptimal for dengue transmission, thus the reduction of risk even in longer lags.

4.	Introduction: effect modification by socio-economic factors – The key premise of the paper is examining whether the climate-dengue relationship is stronger among certain vulnerable sub-populations. In the introduction, the authors should provide more literature around why it is reasonable to hypothesize that climate-disease relationships are stronger among certain sub-populations. Even if it is not available for dengue, there is literature for other climate-health outcomes (e.g., for heat-mortality relationship) to show that disadvantaged communities have lower adaptive capacity and/or are more susceptible to negative health outcomes when exposed to environmental hazards.

Known effects of socio-economic factors have been added to the introduction of the manuscript, as suggested and seen in Lines 80-83: “Certain vulnerable sub-populations have been observed to have higher infectious disease risk incidence such as those with higher poverty incidence, urban to population ratios, and smaller government health expenditures, as they have lower adaptive capacity and are more susceptible to negative health outcomes when exposed to environmental hazards.”

5.	Results: effect modification by socio-economic factors – As shown in Figure 5, I find it curious why the temperature-response relationship is highly uncertain for one SES axes of comparison, but not the other. Do the authors understand why there would be substantial uncertainty in the temperature-response relationship among provinces with low population density, say, but not high population density? The high uncertainty on the poverty incidence comparison could also be leading to the null finding of effect medication, as the temperature-response curves are quite different for the two, but there is so much uncertainty around the high poverty incidence line. As an alternative model specification to the meta-analysis, authors may consider interacting the crossbasis term in the national model (equation 2) by the dichotomous SES variable of interest. Doing so would also give the benefit of being able to interpret the SES variable as a main effect (see point 7).

Thank you for your insightful comment and suggestion. The uncertainty may potentially be relative to the group or strata as what the reviewer mentioned. In response to this suggestion/comment, we have decided to update our model using an interaction analysis instead of the previous stratification model. However, in order to implement the interaction model through DLNM, we needed to go away with the two stage analysis strategy employed previously, and instead implemented a random effects model. A two-stage model and a random effects model are similar in certain respects, this is discussed elaborately with examples by Viechtbauer W. (2022) in the following link: <https://www.metafor-project.org/doku.php/tips:two_stage_analysis>.

Previously, we implemented a two-stage analysis, whereby we estimated the model coefficients and the (co)variance matrix structure per province; a total of 61 combinations of model coefficient and (co)variance. We subsequently pooled this using a meta-analysis framework in the second stage. Only in the second stage analysis did we use the area-specific meta-predictors.

First Stage:

Yt ~ Negative Binomial

Yt ~ alpha + f(cb) + covariates

Second stage:

(coef,covar) ~ 1|Province

With the two stage approach, we will not be able to implement the interaction term since there is no variability with the effect modifier since it is measured at the province level; example shown in the dataframe below.

| Province | Date | Dengue cases | Temperature | Effect modifier (Population density) |
| --- | --- | --- | --- | --- |
| Province 1 | 2020-01-01 | 5 | 25.5 | 3000 |
| Province 1 | 2020-01-02 | 10 | 27.8 | 3000 |
| Province 1 | 2020-01-03 | 30 | 27 | 3000 |
| Province 1 | 2020-01-04 | 5 | 25 | 3000 |

In order to conduct the interaction analysis, we needed to utilize the whole dataset without the first stage analysis. This was done using the GAMM with (Province) random effects, to also estimate the pooled association and through the dataset structure found immediately below.

Yt ~ Negative Binomial

Yt ~ alpha + f(cb)*metapredictor + covariates + random(1|Province)

| Province | Date | Dengue cases | Temperature | Effect modifier (Population density) |
| --- | --- | --- | --- | --- |
| Province 1 | 2020-01-01 | 5 | 25.5 | 3000 |
| Province 1 | 2020-01-02 | 10 | 27.8 | 3000 |
| Province 1 | 2020-01-03 | 30 | 27 | 3000 |
| Province 1 | 2020-01-04 | 5 | 25 | 3000 |
| Province 2 | 2020-01-01 | 25 | 27 | 4000 |
| Province 2 | 2020-01-02 | 1 | 20 | 4000 |
| Province 2 | 2020-01-03 | 3 | 22 | 4000 |
| Province 2 | 2020-01-04 | 51 | 26 | 4000 |

However, in terms of implementing this in R using DLNM, the main effects are not directly included in the model (please refer to the following: <https://github.com/gasparrini/2015_gasparrini_EHP_Rcodedata> ; <https://github.com/gasparrini/CTS-smallarea>). While it would seem intuitive to multiply the interaction term with the crossbasis to get both the main and interaction terms, however, its implementation takes an extra step to do so. In brief, we first identified the comparator or reference percentile of comparison. In this study, we utilized the 5^th^ and 95^th^ percentiles to represent the low and high values of the effect modifier. We utilized the codes from Gaspparini A. (2022) for this analysis.

Let’s take the case of Population density as a working example. We first took the 5^th^ and 95^th^ percentile values for this effect modifier to represent the low and high percentiles. The respective values of population density were then deducted from the 5^th^ or the 95^th^ percentile values, which effectively centers these values around the 5^th^ or 95^th^ percentiles. The temperature crossbasis term is then multiplied by the centered population density in either (low or high) percentiles. The resultant crossbasis represents the exposure-lag-response dependency corresponding to the centering point of the effect modifier (Gasparrini et al., 2015). So, in principle we have two models, one for each effect modifier percentile.

For 5^th^ percentile:

Yt ~ Negative Binomial

Yt ~ alpha + f(cb)+f(cbint05)+gamma_Population density + covariates + random(1|Province)

For 95^th^ percentile:

Yt ~ Negative Binomial

Yt ~ alpha + f(cb)+f(cbint95)+lambda_Population density + covariates + random(1|Province)

In either of the percentiles, the main effects of the effect modifier are identical. We have added a Supplementary Table 3 for the main effects of the effect modifier.

6.	Results: effect modification by socio-economic factors – In the results (line 179-184), the authors should go beyond saying which factors were effect modifiers, and describe how temperature-dengue relationships vary by SES strata. For instance, the authors could describe how dengue incidence within provinces with low healthcare expenditures is highly sensitive to changes in temperature, while dengue incidence provinces with high healthcare expenditures is less sensitive.

This suggestion has been acknowledged by the authors and has been added to the manuscript, as advised, and can be seen in the following lines:

Lines 80-83: “Certain vulnerable sub-populations have been observed to have higher infectious disease risk incidence such as those with higher poverty incidence, urban to population ratios, and smaller government health expenditures, as they have lower adaptive capacity and are more susceptible to negative health outcomes when exposed to environmental hazards”

7.	Discussion: effect modification by socio-economic factors – In the discussion (lines 208-255), the authors should take care not to confuse interpretation of effect modifiers and main effects. For instance, the results provided do not say anything about the relationship between poverty and dengue incidence, and thus do not contrast with existing literature finding an association between poverty and dengue. Poverty incidence would have to be included as a main effect in the model in order to make that comparison. Instead, poverty incidence was not found to be a significant effect modifier of the temperature-dengue association (although, see point 5 about uncertainty).

Please see response in point 5.

8.	Additional model confounders: while the inverted U-shaped relationship with -temperature makes sense, I am curious if the authors considered adjusting for precipitation as a confounder, as precipitation is known to influence dengue, and also be associated with temperature.

Sensitivity analyses adjusting for precipitation derived from ERA5-land showed highly similar risk function, suggesting little to no effect of rainfall on the tempreature-dengue association. The results of the sensitivity analysis has been added to the supplementary materials seen in Figure S4. We did consider adjustment for rainfall but there was no significant difference between the crude model; thus, we opted for the crude model.

Additionally, I have some minor concerns that may improve the readability of the manuscript:

Abstract:

-	“role of socio-economic on the temperature-dengue association remains unclear” could be phrased better (e.g., “heterogeneity in the temperature-dengue relationship across axes of socio-economic vulnerability is not well described.”

-	“explore the modification role by socioeconomic factors” could be edited to “explore effect modification by socioeconomic factors”

-	Keep consistent socio-economic vs. socioeconomic

-	Line 34: “analysis in examining” change to “analysis to examine”, change “explain” to past tense

-	Line 35: “nation wide heterogeneity” – do you mean “heterogeneity between provinces”?

All these suggestions have been reflected in the manuscript in the following lines:

Line 25

Line 27

Line 35

Line 36

Introduction

-	Line 55 – italicize vector name

-	Line 56 – is the 30-fold increase globally?

-	Line 65 – change “higher” to “high”

-	Lines 74 – 78: are these relationships inverse U-shaped?

-	Line 82: add bolded text “linear association between temperature and dengue”

All these suggestions have been reflected in the manuscript in the following lines:

Line 62

Line 63

Line 71

Line 78-79 & 81-82: These studies observed an increase of dengue risk within a certain range of high temperatures. Similar inverse U-shape association was seen in a study by Wu, et. al. These clarifications have been added to the manuscript.

Lines 96-97

Methods

-	Line 97: change to “and were thus excluded from the current study”

-	Line 99: what is the spatial resolution of ERA5-land? Also cite ERA5-land data

-	Line 102: remove word “significantly”

-	Line 104: add “Over the same period,…”

-	Line 114: I would describe this as a DLNM with a negative binomial distribution on the outcome rather than a negative binomial regression coupled with a DLNM

-	Add equation numbers to equations 1 and 2

-	Lines 124-126: what kind of splines were used?

-	Line 129 and Equation 1: I believe the term should be Y­_(t-1,i) instead of Y_(t, i-1). Also the error term symbol is missing in line 129 on my version.

-	Lines 135 – 136: Is the pooled association obtained through equation 2 or by using meta-analysis to pool?

-	Line 136: Instead of “heterogeneity in the pooled association”, do you mean “heterogeneity in the province-specific associations”?

-	Line 138 – 140: citations needed for the packages

All these suggestions have been reflected in the manuscript in the following new line numbers:

Line 111

Line 114

Line 122

Line 123

Line 136

Lines 140 & 161

Removed from the methodology (due to the change of method)

Line 138

Removed from the methodology (due to the change of method)

Line 163

Lines 166-167

Results

-	Line 147: Is the median and range for cases per year? Per week?

-	Lines 146 – 150: Only report the ranges and medians for the 61 provinces

-	Line 148: can get by with fewer decimals on temperatures

-	Figure 1: In the downloadable figure, it looks OK, but in the in-text figure, A and B panels are different sizes. Ensure they are similarly sized.

All these suggestions have been reflected in the manuscript in the following lines:

Line 173: mean, weekly

Line 173-175: We have only reported the dengue incidence median and range.

Line 175: Temperature numbers have been rounded off to the nearest hundredths

Line 415: The figures have been resized to be the same size.

Discussion

-	Line 191: The text here says 27.1 but earlier it said 27.2

-	Line 221: “It’s vectors” – name the vectors

-	Limitations: could some of the SES variables studied affect reporting? For instance, some discussion could be provided around the fact that areas with higher poverty may be less likely to report dengue incidence, thus biasing relationships towards the null

-	Line 261: “decentralized” may be a better term than “devolved”

All these suggestions have been reflected in the manuscript in the following lines:

Line 209: Changed to 27.1, which is the correct result

Line 302: Added vectors as suggested

Line 355-357

Line 345

—

Review for “Socio-economic factors and its influence on the association between temperature and dengue incidence in 61 Provinces of the Philippines”.

This paper used DLNM to identify the association between temperature and dengue incidence by each of the 61 provinces of the Philippines. The paper then conducted meta regression to identify how socioeconomic conditions (as meta regressors) modified these associations. Overall, I think the manuscript is well written and the research design is fine.

Here are some additional comments that the authors may want to address:

1. Line 113, the two-stage analysis (i.e. provincial DLMN + meta regression) is self-coherent. However, I don’t see the need for analyzing the nationwide temperature-dengue associations. It might be misleading for the readers to think that an analysis using national-level time-series of temperature and dengue cases was conducted.

We thank the reviewer for the comment. We have tweaked the analysis with regard to the suggestion from one of the reviewers regarding the usage of an interaction analysis instead of a stratified analysis. By doing so, some of the results changed, however, the overall message remains the same: all of the effect modifiers were able to modify the association between temperature and dengue.

With regard to the query about nationwide temperature-dengue associations, the word may have been indeed misleading. Instead, we used a “nationally-representative temperature-dengue association” to account for the fact that the 61 provinces, may, in principle, be representing the nationwide association to a limited extent.

2. The choice of certain modeling parameters, such as df=4 for temperature, needs to be justified. In addition, while the authors justified the 18-week maximum time lag based on sensitivity analysis (Figure S2), whether such a lag was supported by the disease process can be explained in the manuscript.

Longer lag associations have been observed in other studies and have been added to the manuscript in Lines 225-236.

3. Line 147, is the “median of 23, and interquartile range of 57” for cases per epiweek?

The clarification of “per epiweek” has been added to the manuscript in Lines 167-169.

**4.** Line 171, the meaning of I2 and how it determines the heterogeneity can be explained in the manuscript.

With the change from the two-stage to Generalized Additive Mixed Model (GAMM) in pooling the results, I2 value may not be anymore relevant to this case. However, for clarification purposes, I2 statistic describes the percentage of variation across studies that is due to heterogeneity rather than chance (Higgins and Thompson, 2002; Higgins et al., 2003). I² = 100% x (Q-df)/Q. I² is an intuitive and simple expression of the inconsistency of province-specific associations. For example, a meta-analysis with I2=0 means that all variability in effect size estimates is due to sampling error within studies. On the other hand, a meta-analysis with I2=50 means that half of the total variability among effect sizes is caused not by sampling error but by true heterogeneity between studies (Huedo-Medina et al., 2006).

References:

Higgins JPT, Thompson SG. Quantifying heterogeneity in a meta-analysis. Statistics in Medicine 2002;21:1539-1558.

Higgins JPT, Thompson SG, Deeks JJ, Altman DG. Measuring inconsistency in meta-analyses. British Medical Journal 2003;327:557-560.

Huedo-Medina, T. B., Sánchez-Meca, J., Marín-Martínez, F., & Botella, J. (2006). Assessing heterogeneity in meta-analysis: Q statistic or I² index?. *Psychological methods*, *11*(2), 193.

5. Line 203-204, the statement that 11 weeks accounts for the full cycle of the disease process from mosquito hatching to incubation in infected humans needs some reference.

Longer lag associations have been observed in other studies and have been added to the manuscript in Lines 225-236.

**6.** Line 205, “countries” should be “locations”, as Bangkok is not a country.

This has been acknowledged and the manuscript has been modified accordingly in Line 232.

**7.** Figure 3, I found it interesting that all the provincial temperature-relative risk curves indicated RR=1 at temperature=25 degrees C. Is there any explanation for this?

The temperature at 25 degrees is set as the reference value for the previous two stage analysis, currently the reference value is at 22 degrees Celsius with the Generalized Additive Mixed Model (GAMM). When we set the reference temperature at 25 (or 22), it means that the RR at that point is equal to 1, or assumed as no risk. This is just for part of the parameter specification purposes for interpretability. Because the association tends to be non-linear, the increase is interpreted relative to a reference temperature (which is assumed to be RR=1).

**8.** Also Figure 3 shows that in some provinces RR decreases as temperature gets warmer till 25 degree C, which is in the contrary to the statement between L169-171. Could the authors address/explain this?

Since the new analysis is now using a Generalized Additive Mixed Model (GAMM) and not anymore, a two-stage analysis, there will be no province-specific results to be compared with.

But for clarification purposes, with reference to the previous Figure 3, the pooled association is shown to be an inverted U, however, some provinces did depict either a linear increase or a U-shaped pattern. In brief, the pooled association (inverted U) is in simple terms, the “average” association that one would expect if we were to combine all the province specific associations together. While there would be provinces that are not inverted U shaped, however, the majority or the average pattern is inverted U, and thus depicting this inverted U in the pooled association.

**9.** Lastly, such a two-stage analysis is also used (quite sometimes) in temperature-mortality studies (e.g. Bakhtsiyarava, M., et al.. (2023). Modification of temperature-related human mortality by area-level socioeconomic and demographic characteristics in Latin American cities. Social Science & Medicine, 115526.). The authors could provide such context in the manuscript.

This reference has been cited in our methods in the introduction. In particular, highlighting the fact that such modifications are not just innate to the temperature-dengue association, but are also more apparent in studies conducted in similar literatures of temperature and mortality.
