# Supplementary material for: Socio-economic factors and its influence on the association between temperature and dengue incidence in 61 Provinces of the Philippines, 2010–2019
Source: PLoS Negl Trop Dis. 2023 Oct 23;17(10):e0011700. doi: 10.1371/journal.pntd.0011700 (PMC10621993; doi:10.1371/journal.pntd.0011700)
Supplement: Attachment — Submitted filename: PlosNTDs_dengue_temp_SES_rev2.docx [file pntd.0011700.s014.docx]

In this study, authors examine how various socio-economic factors (including poverty, urbanization, and health-care expenditures) modify the temperature-dengue incidence across provinces in Philippines. To test the effect modification, they follow appropriate methods interaction of the temperature cross basis term. This research question is of importance, and the DLNM model is a good approach for this question. My original concerns have largely been addressed, and the authors have provided a very thorough revision. I have a few remaining recommendations for revisions:

1. **Figure 4**: the figure that is uploaded individually and in the revision looks correct, and has lag as the x-axis. The figure included in the end of the MS text has the wrong x-axis.
2. **Results:** For Figure 4 and in the results line 211-214 of the revised text, please state what the reference level is. In other words, this is the lag response relationship for increases in temperature from what reference percentage to the 90^th^ percentile?
3. **Results:** While the temperature-incidence curves stratified by the SES variables look better under this parametrization of the model, I am a little unsure what to make of the curves stratified by poverty incidence. Do the authors have a theory why the exposure-response relationship should be U-shaped? The magnitude of the main effect on poverty incidence in Table S3 also suggests to me that the model fit may be unstable, or a variable needs to be rescaled.
4. **Discussion:** In a few places, the authors should be more precise with the interpretation of interaction terms versus main effects. For example:
   a. Line 254: “higher population density also led to higher risk in dengue”. This doesn’t appear true from Table S3. The statement I believe the author’s intended is “higher population density was associated with a stronger temperature-incidence relationship”, or something similar.
   b. Line 276: “Average household size was found to have higher dengue risk than small households”. This statement appears to be true from Table S3. However, I wonder if the authors were intending to comment on the temperature-incidence relationship.
   c. Line 298: Same comment for higher health expenditures
   d. **Author summary: “**We also noted that provinces with high population density, less people in urban areas with larger household size, high poverty incidence, higher health spending per capita, and in lower latitudes have higher risk of dengue compared to their counterparts”. Again, I believe the authors intended to comment on the temperature-incidence relationship, and may have meant something like the following: **“**The association between temperature and dengue incidence at high temperatures was stronger among provinces with high population density, less people in urban areas with larger household size, high poverty incidence, higher health spending per capita, and in lower latitudes compared to their counterparts”.
5. **Discussion**: Could higher health expenditures result in higher reporting/health care seeking behavior, which could explain the observed associations?
