# Supplementary material for: Socio-economic factors and its influence on the association between temperature and dengue incidence in 61 Provinces of the Philippines, 2010–2019
Source: PLoS Negl Trop Dis. 2023 Oct 23;17(10):e0011700. doi: 10.1371/journal.pntd.0011700 (PMC10621993; doi:10.1371/journal.pntd.0011700)
Supplement: Attachment — Submitted filename: response to reviewer.docx [file pntd.0011700.s015.docx]

**Overall response**: We thank the reviewer for taking time to read through the revised version of the manuscript. We have provided the respective responses to the queries of the reviewer as shown in the succeeding text.

In this study, authors examine how various socio-economic factors (including poverty, urbanization, and health-care expenditures) modify the temperature-dengue incidence across provinces in Philippines. To test the effect modification, they follow appropriate methods interaction of the temperature cross basis term. This research question is of importance, and the DLNM model is a good approach for this question. My original concerns have largely been addressed, and the authors have provided a very thorough revision. I have a few remaining recommendations for revisions:

**Response**: We appreciate the reviewer’s thorough comments since it has further improved the clarity of the manuscript. We have also addressed the respective comments in point-per-point basis.

1. **Figure 4**: the figure that is uploaded individually and in the revision looks correct, and has lag as the x-axis. The figure included in the end of the MS text has the wrong x-axis.

**Response**: We have included the right figure at the end of the manuscript in response to this comment.

2. **Results:** For Figure 4 and in the results line 211-214 of the revised text, please state what the reference level is. In other words, this is the lag response relationship for increases in temperature from what reference percentage to the 90^th^ percentile?

**Response**: we have included the following to the “title” of Figure 4 and in lines 195-196 which reads “On the other hand, cumulative lag-response association at the 90th percentile, relative to the 50th percentile of the prediction interval…”.

3. **Results:** While the temperature-incidence curves stratified by the SES variables look better under this parametrization of the model, I am a little unsure what to make of the curves stratified by poverty incidence. Do the authors have a theory why the exposure-response relationship should be U-shaped? The magnitude of the main effect on poverty incidence in Table S3 also suggests to me that the model fit may be unstable, or a variable needs to be rescaled.

**Response**: In several studies, most of the association between temperature and dengue were noted to be inverse U-shaped (Xiang et al., 2017, Kakarta et al., 2019). In particular, Xiang et al. (2017) noted that the range from 21.6-32.9degC is suitable for dengue transmission. We did observe a U-shaped risk curve for the main association and in the low poverty incidence association. However, this was not the case for the high poverty incidence association. There seems to be no literature that supports how poverty incidence shapes the risk function. However, we provide a potential reason, which is merely a speculation, why such risk function is inherent for the high poverty incidence areas and not for the low incidence areas.

Since the intent of the effect modification is to identify whether there is key difference in the risk function due to an effect modifier, we can simply assume that such difference is because modifier itself. In this case, the effect modifier represents a variety of behavioral and exposure differences of the population.

Resource-poor households/populations tend to work mostly during extreme conditions, including that during low temperatures and high temperatures in contrast to those resource-rich households/populations. Populations in resource-poor locations would prioritize more food and other basic necessities than personal risk prevention of dengue (e.g. mosquito repelling lotion, long sleeves, etc.). As mentioned above, dengue transmission may persist in a range of 21.6-32.9degC. If we chart against Figure 4 (poverty incidence panel), we note that the range 21 to 29 degC, which is well within the range of dengue transmission, hence the potential high risk at these extremes for the high poverty incidence populations. The low risk at the middle may also be similarly related to low exposure of the population since these are temperatures that are conducive for most less labor-intensive jobs, whereby these populations don’t engage with. These are mostly assumptions that remain to be elucidated in future studies.

As for the scaling of the data, since this is already a rate, measured as a “Proportion of people living under the poverty line per 100,000 people in each province yearly between 2010-2019”, so in principle this is also scaled from 0 to 100%.

4. **Discussion:** In a few places, the authors should be more precise with the interpretation of interaction terms versus main effects. For example:
   a. Line 254: “higher population density also led to higher risk in dengue”. This doesn’t appear true from Table S3. The statement I believe the author’s intended is “higher population density was associated with a stronger temperature-incidence relationship”, or something similar.

**Response**: Thank you for the comment. We have rephrased the following to: “higher population density was associated with a visually higher temperature-incidence association than low population dense areas“.

b. Line 276: “Average household size was found to have higher dengue risk than small households”. This statement appears to be true from Table S3. However, I wonder if the authors were intending to comment on the temperature-incidence relationship.

**Response**: This is noted. We are afraid that the limited evidence restricts the further extrapolation to the mechanisms behind how large household sizes have different and steeper dengue risks at both ends of the temperature range than small household sizes. However, we believe that this may be remotely related to the exposure and behavioral differences between large and small sized households, similar the assumption we set for poverty incidence. But this can also be an effect of high reporting/health care seeking behavior as mentioned in response in Query 5. The larger the households, the more the family members, the higher the rate of reporting. However, all of these are speculations.

c. Line 298: Same comment for higher health expenditures

**Response**: Please see response in Query 5 (below).

d. **Author summary: “**We also noted that provinces with high population density, less people in urban areas with larger household size, high poverty incidence, higher health spending per capita, and in lower latitudes have higher risk of dengue compared to their counterparts”. Again, I believe the authors intended to comment on the temperature-incidence relationship, and may have meant something like the following: **“**The association between temperature and dengue incidence at high temperatures was stronger among provinces with high population density, less people in urban areas with larger household size, high poverty incidence, higher health spending per capita, and in lower latitudes compared to their counterparts”.

**Response**: Thank you for the suggestion and apologies for the inconsistencies. We have reflected this author summary.

5. **Discussion**: Could higher health expenditures result in higher reporting/health care seeking behavior, which could explain the observed associations?

**Response**: Thank you for the insight. Indeed, this may be one of the potential reasons why higher expenditure could lead to higher reporting/health care seeking behavior, thus may potentially lead to steeper temperature-dengue association in high health care spending locations than those low health care spending ones. We have included this potential reason in the manuscript in lines 299 - 300.

References:

- Kakarla, S., Caminade, C., Mutheneni, S., Morse, A., Upadhyayula, S., Kadiri, M., & Kumaraswamy, S. (2019). Lag effect of climatic variables on dengue burden in India. Epidemiology & Infection, 147, E170. doi:10.1017/S0950268819000608
- Xiang J, Hansen A, Liu Q, Liu X, Tong MX, Sun Y, et al. Association between dengue fever incidence and meteorological factors in Guangzhou, China, 2005–2014. Environmental Research. 2017;153:17-26.
